# Prevalence of mental health problems in frontline healthcare workers after the first outbreak of COVID-19 in China: a cross-sectional study

**DOI:** 10.1186/s12955-021-01743-7

**Published:** 2021-03-22

**Authors:** Wen-Ping Guo, Qing Min, Wei-Wei Gu, Liang Yu, Xiao Xiao, Wei-Bing Yi, Hong-Liang Li, Bei Huang, Jun-Li Li, Ya-Jun Dai, Jian Xia, Jie Liu, Bei Li, Ben-Hong Zhou, Minglun Li, Hong-Xi Xu, Xuan-Bin Wang, Wen-Yuan Shi

**Affiliations:** 1 Renmin Hospital at Intensive Care Unit, Emergency Room, Laboratory of Chinese Herbal Pharmacology of Oncology Center, and Department of Traditional Chinese Medicine; Hubei Key Laboratory of Wudang Local Chinese Medicine Research; Biomedical Research Institute; School of Basic Medicine, Hubei University of Medicine, Shiyan, 442000 Hubei Province China; 2 Shuguang Hospital; School of Pharmacy, Shanghai University of Traditional Chinese Medicine, 1200 Cailun Road, Shanghai, 201203 China; 3 School of Pharmacy, Hubei University of Science and Technology, 88 Xianning Road, Xianning, 437100 Hubei Province China; 4 Shiyan Center for Disease Prevention and Control, 86 Tianjin Road, Shiyan, 442000 Hubei Province China; 5 Department of Pharmacy and Department of Spleen-Stomach, Xiaogan Hospital of Traditional Chinese Medicine, 249 Huaiyin Road, Xiaogan, 432000 Hubei Province China; 6 Xiangyang Hospital, Hubei University of Medicine, 15 Jiefang Road, Xiangyang, 441000 Hubei Province China; 7 Emergency Center, Zhongnan Hospital, Wuhan University, 169 Donghu Road, Wuhan, 430071 Hubei Province China; 8 Department of Pharmacy, Renmin Hospital, Wuhan University, 99 Zhangzhidong Road, Wuhan, 430060 China; 9 Department of Radiation Oncology, University Hospital, LMU Munich, Marchioninistraße 15, Munich, 81377 Germany

**Keywords:** Coronavirus disease 2019, Healthcare workers, Mental health outcome, Anxiety, Insomnia, Depression, Post-traumatic stress disorder

## Abstract

**Background:**

More than 210,000 medical workers have fought against the outbreak of Coronavirus Disease 2019 (COVID-19) in Hubei in China since December 2019. However, the prevalence of mental health problems in frontline medical staff after fighting COVID-19 is still unknown.

**Methods:**

Medical workers in Wuhan and other cities in Hubei Province were invited to participate a cross-sectional and convenience sampling online survey, which assessed the prevalence of anxiety, insomnia, depression, and post-traumatic stress disorder (PTSD).

**Results:**

A total of 1,091 responses (33% male and 67% female) were valid for statistical analysis. The prevalence was anxiety 53%, insomnia 79%, depression 56%, and PTSD 11%. Healthcare workers in Wuhan were more likely to face risks of anxiety (56% vs. 52%, *P* = 0.03) and PTSD (15% vs. 9%, *P* = 0.03) than those in other cities of Hubei. In terms of educational attainment, those with doctoral and masters’ (D/M) degrees may experience more anxiety (median of 7.0, [interquartile range (IQR) 2.0–8.5] vs. median 5.0 [IQR 5.0–8.0], *P* = 0.02) and PTSD (median 26.0 [IQR 19.5–33.0] vs. median 23.0 [IQR 19.0–31.0], *P* = 0.04) than those with lower educational degrees.

**Conclusions:**

The mental problems were an important issue for the healthcare workers after COVID-19. Thus, an early intervention on such mental problems is necessary for healthcare workers.

**Supplementary Information:**

The online version contains supplementary material available at 10.1186/s12955-021-01743-7.

## Background

Since the first outbreak of the coronavirus disease 2019 (COVID-19) pandemic in late December, 2019 [1], 82,933 cases have been confirmed, and 4633 people died in mainland China up till May 14th, 2020 [2]. In Hubei Province, especially Wuhan, the capital city of Hubei that was the epicenter of the COVID-19 outbreak in mainland China, there were 50,339 confirmed cases (60.69% of mainland China), and 3869 patients died (83.51% of mainland China) [3].

More than 170,000 first-line medical workers have joined the battle against COVID-19 [4]. Furthermore, 45,322 medical workers from other provinces have been recruited to help reduce the pressure on healthcare personnel in Wuhan. Among them, 38,478 (84.90%) doctors and nurses have worked alongside their Wuhan local colleagues [5].

The extreme exposure due to their profession, a great number of cases, dying patients, shortage of personal protection equipment, having to put on heavy isolation suits as well as the lack of effective drugs and treatment strategies has put all medical staff at a high risk of infection and even death. They were also overworked and had to reduce contact with their families [6–8]. Thus COVID-19 may induce mental stress including anxiety, insomnia, depression, and post-traumatic stress disorder (PTSD) [9], all of which lasts for even a long time [10]. Nevertheless, there was no investigation on the incidence of mental health negative outcomes in healthcare workers after the first wave of COVID-19 in Hubei. Thus, in this study, we launched an online survey on mental health of medical workers fighting COVID-19 in Wuhan and other cities of Hubei, aiming to provide basic data for intervention with regards to medical workers’ mental health.

## Methods

### Study design and participants

The study was a cross-sectional, hospital-based and convenience sampling survey via a multi-region-stratified sampling from May 15th to 31st, 2020. During the days, there were no new COVID-19 cases in Hubei [11]. The subjects were involved with the medical workers in three hospitals in Wuhan, Jinyintan Hospital (most of professionals were from other provinces), Renmin Hospital of Wuhan University, and Zhongnan Hospital of Wuhan University, and in five hospitals of other cities of Hubei Province, Xianning Center Hospital, Xiaogan Hospital of Traditional Chinese Medicine, the Second Jingmen Hospital, Xiangyang Hospital and Renmin Hospital of Hubei University of Medicine. All these hospitals had been treated the patients since the COVID-19 outbreak. Physicians, nurses and other professionals (including radiology technicians, pharmacists, clinical laboratory technicians, disease prevention and control department staff, ambulance drivers, and administrative staff) at the frontlines to fight the COVID-19 crisis in Hubei including Wuhan had participated in the survey via the Quick Response (QR) code of Questionnaire Star based on the social software, WeChat App. (Tencent Inc., Shenzhen, China) [12]. 1805 medical workers in 18 Wechat groups in eight hospitals were invited with a goal of at least 60% response rate (at least 1083 respondents completed questionnaires). Then the respondents were screened according to the inclusion and exclusion criteria. The inclusion criteria were as follows: (1) Medical staff who had the initial fight of COVID-19 in Hubei for one month; (2) Medical staff from other provinces who have been on the frontiers during the COVID-19 outbreak in Hubei; (3) Staff without mental conditions before fighting COVID-19; and (4) Staff without severe diseases of the heart, liver, kidney, or blood system. The exclusion criteria were as follows: (1) Worked in other provinces to fight COVID-19, excluding Hubei; (2) Were medical staff in Hubei but did not work during the COVID-19 outbreak; and (3) Disagreed to grant authorization to use their information or filled an invalid questionnaire. This study was approved by the Ethics Committee of Renmin Hospital of Hubei University of Medicine. All participants were required to clarify that they understood the questionnaire. They were then asked to sign online informed consent that allowed the investigators to use their information; if not, their answers were identified as adhering to the exclusion criteria.

### Participants’ personal information

The structured online questionnaire consisted of personal information that could be potential influencing factors: sex, age, marital status, education level, occupation, and working location. The survey was conducted from May 15th to 31st, 2020. As Wuhan was the most severely affected, the sampling was done from Wuhan and other cities in the Hubei Province to compare the difference between the effects of the pandemic in the frontline city and other second-line cities of Hubei.

### Measurement of anxiety

To measure the severity of self-reported anxiety, the Generalized Anxiety Disorder-7 (GAD-7) assessment scale was used [13]. The anxiety levels were assessed using a self-assessment questionnaire ranging from zero to three. The higher the score on the questionnaire, the more the anxiety potentially faced. Briefly, the total score was interpreted as normal (0–4), mild (5–9), moderate (10–14), and severe (15–21). The cutoff score for detecting symptoms of major anxiety was ten based on Lai et al., 2020 [13]. In this study, the Cronbach’s alpha coefficient of the GAD-7 was 0.95.

### Measurement of Insomnia

Highly intensive work and excessive stress may lead to insomnia. To investigate whether participants were suffering from insomnia and to what degree they experienced it after one month of fighting COVID-19, the assessment of sleep quality and disturbances was conducted using the Pittsburgh Sleep Quality Index (PSQI) [14]. The severity of insomnia was set at levels ranging from zero to three in each item. The total score was presented as normal (0–7), subthreshold (8–14), moderate (15–21), and severe (22–28) insomnia. Poorer quality of sleep was associated with a higher score and identified as scores ranging from 15 to 28 [13]. The cutoff score for detecting symptoms of insomnia was seven based on Lai et al., 2020 [13]. The Cronbach’s alpha coefficient in the PSQI was 0.87.

### Measurement of depression

To measure the potential depression among medical staff, the Patient Health Questionnaire (PHQ-9) was used for assessment [15]. Each item ranged from zero to three. The total score was interpreted as normal (0–4), mild (5–9), moderate (10–14), and severe (15–21) depression. The cutoff score for depression was 10 based on Lai et al., 2020 [13]. The Cronbach’s alpha coefficient was 0.92.

### Measurement of PTSD

To assess the potential of the post-traumatic stress syndrome, the PTSD Checklist-Civilian Version (PCL-C) was conducted as a self-reported online survey [14]. Each item represented the level of a particular symptom, rated on a five-point Likert scale from one (not at all) to five (extremely). The total scores range from 17 to 85. Higher scores indicated more severe PTSD symptoms. The severity from normal to severe was 17–37 (normal), 38–49 (mild), and 50–85 (severe). The cutoff score for PTSD was 37 based on Liu et al. [14]. In the present study, the Cronbach’s alpha coefficient of the PCL-C was 0.95.

### Statistical analysis

All statistical analyses were conducted using the SPSS Statistical Software version 25 (IBM Corp)*.* The significance level was set as *P* value < 0.05. All tests were two-tailed [13]. Since the original scores of the measurement tools were not normally distributed, they were presented as medians with interquartile ranges (IQRs) [13]. The ranked data for anxiety [13], insomnia [14], depression [15], and PTSD [14] were presented as numbers and percentages. The severity of each symptom between two or more groups was compared using the nonparametric Mann–Whitney *U* test and Kruskal–Wallis test [13]. Subsequently, the binary and multivariable logistic regression analysis was conducted to determine potential risk factors for anxiety [13], insomnia [14], depression [15], and PTSD [14] symptoms, and the associations between risk factors and results were presented as odds ratios (ORs) and 95% CIs after adjustment for confounders, including sex, age, marital status, educational level, occupation, and working location [13].

## Results

### Demographic characteristics

1151 out of 1805 medical workers from 8 hospitals participated in the survey and the response rate was 63.8%. Among them, 1091 (95%) responses were considered as valid for statistical analysis including those from physicians (202, 19%), nurses (554, 51%), and other healthcare workers (335, 31%). The sex, occupational and location data in nonrespondents were similar to those in respondents (Additional file 1: Table S1). The 60 (5%) discarded responses were those from participants who disallowed the use of their information for research (10 persons, 1%) and those who filled invalid ages (50 persons, 4%)—they filled their either sex or names instead of a number. Regarding the working location, it was found that the healthcare staff dominantly worked in other cities of Hubei Province rather than Wuhan. The demographics of the respondents are listed in Table 1.

**Table 1:** Demographics of the respondents (n = 1091)

	No. (%)				
		Location		Education	
Characteristic	Total	Wuhan	Other cities	D/M	U/H
Overall	1091 (100)	353 (32)	738 (68)	117 (11)	974 (89)
Sex					
Male	356 (33)	120 (34)	236 (32)	52 (44)	304 (31)
Female	735 (67)	233 (66)	502 (68)	65 (56)	670 (69)
Age, years					
< 25	107 (10)	70 (20)	37 (5)	0 (0)	107 (11)
25–45	781 (72)	196 (56)	585 (79)	101 (86)	680 (70)
> 46	203 (18)	87 (25)	116 (16)	16 (14)	187 (19)
Marital status					
M/C	837 (77)	212 (60)	625 (74.67)	96 (82)	741 (76)
S/D/W	254 (23)	141 (40)	113 (44.49)	21 (18)	233 (24)
Occupation					
Physician	202 (19)	107 (30)	95 (13)	72 (62)	130 (13)
Nurse	554 (51)	213 (60)	341 (46)	5 (4)	549 (56)
Other staff	335 (31)	33 (10)	302 (41)	40 (34)	295 (30)

M/C: married/cohabitating; S/D/W: single/Divorced/widowed; D/M: Doctor/master; U/H: undergraduate (college)/High school ((including special/technical secondary school)

### Scores from measurement tests and affecting factors

Respondents with D/M degrees who worked in Wuhan had higher scores in anxiety and PTSD as compared to those with U/H degrees who worked in other cities in Hubei Province (Table 2).

**Table 2:** Measurement scores of anxiety, insomnia, depression and PTSD measurements in total cohort and subgroups

		Sex			Marital status			Education level			Location			Occupation			
		median (IQR)			median (IQR)			median (IQR)			median (IQR)			median (IQR)			
Scale	Total score, median (IQR)	Male	Female	*P* value	M/C	S/D/W	*P* value	D/M	U/H	*P* value	Wuhan	Other cities	*P* value	Physician	Nurse	Other staff	*P* value
Anxiety	5.0 (0.5–8.0)	5.0 (0.0–8.0)	5.0 (1.0–8.0)	.71	5.0 (0.0–8.0)	5.0 (1.0–8.0)	.96	7.0 (2.0–8.5)	5.0 (5.0–8.0)	.02	6.0 (5.0–8.0)	5.0 (0.0–8.0)	.2	2.0 (0.0–7.0)	1.0 (0.0–4.0)	0.0 (0.0–2.0)	.51
Insomnia	9.0 (6.0–11.0)	8.0 (6.0–11.0)	9.0 (6.0–11.0)	.16	8.0 (6.0–11.0)	9.0 (6.0–11.0)	.42	9.0 (6.0–11.0)	8.5 (6.0–11.0)	.3	9.0 (6.0–11.0)	8.0 (6.0–11.0)	.88	4.0 (3.0–6.0)	6.0 (4.0–8.0)	5.0 (3.0–7.0)	.6
Depression	5.0 (2.0–9.0)	5.0 (2.0–8.0)	5.0 (2.0–9.0)	<.0001	5.0 (2.0–8.0)	5.0 (2.0–9.0)	.75	6.0 (2.0–9.0)	5.0 (2.0–9.0)	.33	5.0 (2.0–9.0)	5.0 (2.0–8.0)	.52	0.0 (0.0–0.0)	2.0 (0.0–4.0)	0.0 (0.0–1.0)	.9
PTSD	23.0 (19.0–31.0)	23.0 (19.0–31.0)	23.0 (19.0–31.0)	.88	23.0 (19.0–31.0)	23.0 (19.0–30.0)	.89	26.0 (19.5–33.0)	23.0 (19.0–31.0)	.04	25.0 (19.0–33.0)	23.0 (18.0–30.0)	.01	17.0 (17.0–19.0)	19.0 (17.0–22.0)	18.0 (17.0–20.0)	.59

PTSD: post traumatic stress disorder; IQR: interquartile range; M/C: married/cohabitating; S/D/W: single/divorce/widow; D/M: doctorial/master’s degree; U/H: undergraduates/high school (including special/technical secondary school) graduates

### Prevalence of measurements and key associated factors

In terms of the differences in education level, participants with doctoral and master’s (D/M) degrees reported more anxiety and PTSD as compared to those who are undergraduates/high school (U/H) (including special/technical secondary school) graduates. Furthermore, healthcare workers in Wuhan were most likely to face risks of anxiety and PTSD than those in other cities of Hubei (Table 3).

**Table 3:** Severity categories of anxiety, insomnia, depression and PTSD measurements in total cohort and subgroups

		Sex			Age					Marriage			Education			Location		
		No. (%)			No. (%)					No. (%)			No. (%)			No. (%)		
Severity Category	Total, No. (%)	Female	Male	*P* value	≤ 25	26 ≤ age ≤ 35	36 ≤ age ≤ 45	≥ 46	*P* value	M/C	S/D/W	*P* value	U/C	D/M	*P* value	Wuhan	Other cities	*P* value
Anxiety																		
Normal	513 (47)	338 (46)	175 (49)	.73	51 (48)	216 (45)	147 (49)	99 (49)	.89	398 (48)	115 (45)	.52	470 (48)	43 (37)	.04	157 (44)	356 (48)	.03
Mild	418 (38)	288 (39)	130 (37)		37 (35)	193 (40)	116 (39)	72 (35)		317 (38)	101 (40)		364 (37)	54 (46)		138 (39)	280 (38)	
Moderate	98 (9)	67 (9)	31 (9)		13 (12)	46 (10)	20 (7)	19 (9)		73 (9)	25 (10)		89 (9)	9 (8)		37 (10)	61 (8)	
Severe	62 (6)	42 (6)	20 (6)		6 (6)	26 (5)	17 (6)	13 (6)		49 (6)	13 (5)		51 (5)	11 (9)		21 (6)	41 (6)	
Insomnia																		
Normal	231 (21)	153 (21)	78 (22)	.10	26 (24)	101 (21)	54 (18)	50 (25)	.20	183 (22)	48 (19)	.46	213 (22)	18 (15)	.40	72 (20)	159 (22)	.70
Mild	524 (48)	344 (47)	180 (51)		62 (58)	220 (46)	147 (49)	95 (47)		401 (48)	123 (48)		465 (48)	59 (50)		175 (50)	349 (47)	
Moderate	279 (26)	194 (26)	85 (24)		13 (12)	137 (28)	84 (28)	45 (22)		211 (25)	68 (27)		249 (26)	30 (26)		84 (24)	195 (26)	
Severe	57 (5)	44 (6)	13 (4)		6 (6)	23 (5)	15 (5)	13 (6)		42 (5)	15 (6)		47 (5)	10 (9)		22 (6)	35 (5)	
Depression																		
Normal	479 (44)	320 (44)	159 (45)	.09	51 (48)	216 (45)	147 (49)	65 (30)	.25	364 (43)	115 (45)	.56	430 (44)	49 (42)	.21	158 (45)	321 (44)	.44
Mild	418 (38)	275 (37)	143 (40)		37 (35)	193 (40)	108 (36)	80 (39)		327 (39)	91 (36)		376 (39)	42 (36)		126 (36)	292 (40)	
Moderate	122 (11)	84 (11)	38 (11)		13 (12)	46 (10)	28 (9)	35 (17)		92 (11)	30 (12)		106 (11)	16 (14)		45 (13)	77 (10)	
Severe	72 (7)	56 (8)	16 (4)		6 (6)	26 (5)	17 (6)	23 (1)		54 (6)	18 (7)		62 (6)	10 (9)		24 (7)	48 (7)	
PTSD																		
Normal	970 (89)	650 (88)	320 (90)	.47	92 (86)	433 (90)	270 (90)	175 (86)	.33	746 (89)	224 (88)	.68	873 (90)	97 (83)	.03	303 (86)	667 (90)	.03
Mild	87 (8)	63 (9)	24 (7)		10 (9)	40 (8)	21 (7)	16 (8)		65 (8)	22 (9)		71 (7)	16 (14)		34 (10)	53 (7)	
Severe	34 (3)	22 (3)	12 (3)		5 (5)	8 (2)	9 (3)	12 (6)		26 (3)	8 (3)		30 (3)	4 (3)		16 (5)	18 (2)	

Severity Category	Occupation			
	Physician	Nurse	Other staff	
	No. (%)	No. (%)	No. (%)	*P* value
Anxiety				
Normal	89 (44)	274 (49)	150 (45)	.98
Mild	84 (42)	205 (37)	129 (39)	
Moderate	17 (8)	51 (9)	30 (9)	
Severe	12 (6)	24 (4)	26 (8)	
Insomnia				
Normal	41 (20)	110 (20)	80 (24)	.59
Mild	93 (46)	279 (50)	152 (45)	
Moderate	52 (26)	137 (25)	90 (27)	
Severe	16 (8)	28 (5)	13 (4)	
Depression				
Normal	89 (44)	250 (45)	140 (42)	.24
Mild	66 (33)	209 (38)	143 (43)	
Moderate	28 (14)	62 (11)	32 (10)	
Severe	19 (9)	33 (6)	20 (6)	
PTSD				
Normal	175 (87)	494 (90)	301 (90)	.5
Mild	20 (10)	39 (7)	28 (8)	
Severe	7 (3)	21 (4)	6 (2)	

PTSD: post traumatic stress disorder; IQR: interquartile range; M/C: married/cohabitating; S/D/W: single/divorce/widow; D/M: doctorial/master’s degree; U/H: undergraduates/high school (including special/technical secondary school) graduates

### Risk factors for mental health

To present the risk factors for mental health negative outcomes clearly, the identified risk factors for mental health negative outcomes using binary and multinomial logistic regression analysis was listed in Table 4. The results showed that the differences in education level and working location attributed to the increased risks of anxiety and PTSD. Healthcare workers with D/M degrees were more anxious compared to those with U/H degrees. Meanwhile, those working in Wuhan, the COVID-19 pandemic epicenter in China, experienced more anxiety as compared to those working in cities other than Wuhan in Hubei Province. Furthermore, people with D/M degrees differed from those with U/H degrees in terms of being most at risk for mild PTSD (Table 4). However, interestingly, unlike the previous studies, sex (female) and occupation (nurses) did not attribute to the high risks of mental health problems for medical workers in our study.

**Table 4:** The identified risk factors for mental health negative outcomes using binary and multinomial logistic regression analysis

	Variable	No. (%)	Adjusted OR (95%)	*P* value
Anxiety				
Sex	Male	51 (15)	1[Reference]	.72
	Female	109 (15)	1.049 (0.804–1.368)	
Education level	D/M	20 (17)	1.625 (1.050–2.516)	.03
	U/H	140 (14)	1 [Reference]	
Location	Wuhan	58 (16)	1 [Reference]	
	Other cities	102 (14)	0.714 (0.530–0.962)	.03
Occupation	Physician	29 (14)	1 [Reference]	
	Nurse	75 (13)	1.049 (0.804–1.368)	.72
	Other staff	56 (17)	0.890 (0.659–1.201)	.79
Insomnia				
Sex	Male	108 (28)	1 [Reference]	
	Female	238 (32)	1.547 (0.934–2.563)	.09
Education level	D/M	26 (23)	1.810 (0.882–3.716)	.11
	U/H	168 (17)	1 [Reference]	
Location	Wuhan	106 (30)	1 [Reference]	.94
	Other cities	230 (31)	0.979 (0.585–1.638)	
Occupation	Physician	68 (34)	1 [Reference]	
	Nurse	165 (30)	1.237 (0.617–2·481)	.55
	Other staff	103 (31)	1.357 (0.776–2.372)	.29
Depression				
Sex	Male	140 (19)	1 [Reference]	
	Female	54 (15)	1.287 (0.902–1.828)	.17
Education level	D/M	26 (23)	0.829 (0.483–1.423)	.5
	U/H	168 (17)	1 [Reference]	
Location	Wuhan	69 (20)	1 [Reference]	
	Other cities	125 (17)	0.950 (0·649–1·391)	.79
Occupation	Physician	47 (23)	1 [Reference]	
	Nurse	95 (17)	0.681 (0.434–1.069)	·1
	Other staff	52 (16)	0.645 (0.397–1.047)	0.8
PTSD (mild)				
Sex	Male	24 (7)	1 [Reference]	
	Female	63 (9)	0.742 (0.448–1.230)	.25
Education level	D/M	16 (14)	2.025 (1.013–4.050)	.046
	U/H	71 (7)	1 [Reference]	
Location	Wuhan	34 (10)	1 [Reference]	.424
	Other cities	53 (7)	1.386 (0.623–3.082)	
Occupation	Physician	20 (10)	1 [Reference]	
	Nurse	39 (7)	0.890 (0.450–1.760)	.74
	Other staff	28 (8)	0.815 (0.467–1.422)	.47

PTSD: post traumatic stress disorder; OR: odd ratio; D/M: doctorial/master’s degree; U/H: undergraduates/high school (including special/technical secondary school) graduates

The data for risk factors in female and male healthcare workers, nurses and physicians were similar. This indicates that one month after fighting COVID-19, female and males healthcare workers, nurses and physicians had similar degrees of mental health negative outcomes (Table 4).

## Discussion

Since COVID-19 attacked Wuhan as well as the Hubei Province in central China in December 2020, more than 170,000 local healthcare workers and 45,322 other provincial medical staff have been in Wuhan and other cities in Hubei to fight against the little-known virus to save as many patients as possible. However, treating patients has put these workers at high risk of viral infection and adverse mental health problems. As the severe acute respiratory syndrome (SARS) was a similar infective pandemic that led to similar mental health conditions for medical workers, it could be used as a source of reference for the situation caused by COVID-19. In this context, a survey involving 80 nurses in Taiwan during the SARS pandemic showed that the incidence of mental health problems involved insomnia (37%), depression (38.5%), and PTSD (33%) [16]. Furthermore, when medical workers were required to quantify their mental health after a year of the SARS epidemic, 16 out of 56 (28.57%) of the staff in Beijing confirmed that they had PTSD [17]. Regarding COVID-19, a previous study reported that in China, the prevalence of anxiety, insomnia, and depression among healthcare workers was 44.6%, 34.0%, and 50.4%, respectively [13]. Meanwhile, in Italy, the prevalence of anxiety, insomnia, and depression for healthcare workers was 19.80%, 8.27%, 24.73%, and 49.38%, respectively [8]. Moreover, a systematic review and meta-analysis showed that the pooled prevalence rate of anxiety ranged from 22.6 to 36.3% and that of insomnia and depression ranged from 38.9 and 16.5–48.3%, respectively[18]. These previous studies suggest that mental health should be paid great attention in healthcare workers. However, after fighting COVID-19, the prevalence of mental health problems for healthcare workers in Wuhan and other cities of Hubei have remained unknown. Thus, in this study, we launched an online survey on the mental health problems of healthcare workers in Wuhan and other cities of Hubei from May 15^th^ to 31^st^, 2020, one month after they had finished fighting COVID-19 with the aim of providing basic data to aid in mental disorder intervention for medical workers after the pandemic. The results showed that 1151 out of 1805 medical workers from 8 hospitals had participated in the survey and the response rate was 63.8%, of which 1091 (95%) answers were available for statistics.

As to the sex of participants, the previous studies showed that female were dominantly 76.7% [13], 77.2% [8] and 69.8% [12]. This may be due to the fact that the majority of healthcare workers were female nurses [13]. Additionally, among 45,322 medical workers from other provinces helping colleagues of Wuhan, nurses were 28,679 (67.8%) [5]. Being in line with the previous reports, 67% respondents were female in our study.

Generally, the incidence rate for mental health problems was anxiety (53%), insomnia (79%), depression (56%), and PTSD (11%), respectively. This prevalence was found to be not less than that during the COVID-19 pandemic [8, 13]. This may indicate that there would be little relief for healthcare workers one month after the first wave of COVID-19 and that more attention should be paid to this issue [19].

Later, in the sub-analysis, our data revealed that the healthcare staff working in Wuhan had higher PTSD scores as compared to their colleagues in other cities of Hubei Province (Tables 2 and 4). This result was consistent with the previous studies on the mental health of healthcare workers who battled the pandemic [8, 13].

Besides working locations, the main difference from previous studies on the key impact factors attributing to mental health problems was educational level. In previous studies, the pivotal impact factors were sex and occupation as female professionals and nurses obtained higher scores in the measurement of mental health symptoms such as anxiety, depression, and PTSD [13, 18, 20]. It has been concluded that nursing staff—most of whom are female—spent more time on caring for patients and subsequently, faced great risks of mental health problems [8, 13, 18]. However, it was interesting to note that there was no significant difference between sex and occupation in this study. This may be a result of the difference in the number of female nurses involved in this study and those in previous studies. In the previous study, there were 90.8% female nurses (694) [13]. However, in our study, only 72% (416) nurses were female. In addition, another potential reason for the difference may be due to the questionnaire and/or the research quality.

Moreover, rather than sex and occupational factors, our study found that the education levels of participants (D/M degrees) had a potential impact on the risk of mental health problems. It has been reported that psychological distress influences the people with higher education because research school career is more difficulty. In addition to the stresses of the pandemic, those with D/M degrees have an already existent drive to be successful [21, 22], which adds to the pressure on them and could have contributed to our findings. However, it should be noted that some researchers disagree with the idea that those with D/M degrees have an inherent need to succeed [23]. Furthermore, educational degree is not significant for depression and insomnia, and only marginally significant for mild PTSD and anxiety using *p* = 0.05 as significance level. Since depression, insomnia and PTSD could be indirect and more severe problems than anxiety, these complicated outcomes may result from sampling bias [20].

To reduce the mental problems, for the individuals, mental health self-help coping strategies may include accessing psychological materials such as books and online messages on mental health [24]. Also, family relationship affects the physical, social, and emotional health of individuals. Thus keeping in touch with family members is a good way to enhances individual adaptation [22]. Furthermore, exercise and positive social activities (including discussing and sharing experience with colleagues) may effectively reduce the milder clinical mood symptoms or sub-threshold syndromes before they evolve to more complex and enduring psychological responses [18].

In addition, social support network should be established to reduce the pressure on healthcare workers, for example, sending more medical staff to reduce work intensity, adopting strict infection control, providing personal protective equipments, offering practical guidance [24], and providing a range of psychological services [24], including training for professionals on identifying risk factors and symptoms of mental distress, and psychotherapy [22].

## Limitations

The survey was convenience sampling, a kind of non-probability, in which the participants had filled the online questionnaire freely. Weighting was not considered during analysis as the sampling design of the survey was non-probability [25]. This may limit to reflect the real ratio between different groups. For example, the sample of staff with D/M degrees was a little number compared with U/H degrees (11% vs. 89%), which needs to be improved in future study. Though the response rate of this study was over 60%, the existence of response bias is still probable mainly because those with severe mental diseases may have difficulty accepting and disclosing emotions and be unwilling to fill the online form [12]. Furthermore, the filling of the questionnaire was carried out in two weeks and lacked longitudinal follow-up.

## Conclusions

The problems of anxiety, insomnia, depression, and PTSD were an important issue for the healthcare workers after COVID-19. Thus, an early intervention on such mental problems is necessary for healthcare workers.

## Supplementary Information

**Additional file 1: Table S1**. Sex, occupation and geographic data of nonrespondents.

## Data Availability

The datasets generated and/or analyzed during the current study are not publicly available because an informed consent was not obtained from the participants during enrollment; however, these datasets are available from the corresponding author at 470,803,618@qq.com on a reasonable request.

## Abbreviations

COVID-19: Coronavirus disease 2019
PTSD: Post-traumatic stress disorder
QR: Quick Response
GAD-7: The Generalized Anxiety Disorder-7
PSQI: The Pittsburgh Sleep Quality Index
PHQ-9: The Patient Health Questionnaire
PCL-C: The PTSD Checklist-Civilian Version
IQRs: Interquartile ranges
ORs: Odds ratios
D/M: Doctoral and master’s
U/H: Undergraduates/high school
NHCPRC: National Health Commission of the People’s Republic of China
HCHB: Health Commission of Hubei Province
