# Supplementary material for: Prevalence of mental health problems in frontline healthcare workers after the first outbreak of COVID-19 in China: a cross-sectional study
Source: Health Qual Life Outcomes. 2021 Mar 22;19:103. doi: 10.1186/s12955-021-01743-7 (PMC7983094; doi:10.1186/s12955-021-01743-7)
Supplement: Supplementary file 1 — Additional file 1: Table S1. Sex, occupation and geographic data of nonrespondents. [file 12955_2021_1743_MOESM1_ESM.docx]

**Supplementary Table S1. Sex, occupation and geographic data of nonrespondents.**

|  | **Total** | **Sex** | | **Occupation** | | | **Location** | |
| --- | --- | --- | --- | --- | --- | --- | --- | --- |
|  |  | **Male** | **Female** | **Physician** | **Nurse** | **Other staff** | **Wuhan** | **Other cities** |
| No, (%) | 654 | 189 (29) | 465 (71) | 150 (23) | 347 (53) | 157 (24) | 229 (35) | 425 (65) |
